# Inadequate fluid replacement during prolonged cycling in the heat impairs time trial performance independent of serum sodium concentrations

**DOI:** 10.1007/s00421-026-06245-2

**Published:** 2026-05-02

**Authors:** Sean A. Sage, Fabiana I. Smaira, Jeffrey W. F. Aldous, Martyn G. Morris, Andrew Blow, Jonny Tye, Michael L. Newell

**Affiliations:** 1 Institute for Sport and Physical Activity Research (ISPAR), University of Bedfordshire, Bedford, MK41 9EA UK; 2 Center of Lifestyle MedicineLaboratory of Assessment and Conditioning in RheumatologyFaculdade de Medicina FMUSP, Hospital das Clínicas HCFMUSP, Universidade de Sao Paulo, Av. Dr. Arnaldo, 455, 3° Andar, São Paulo, SP 01246-903 Brazil; 3 School of Health, Medicine and Life Sciences, University of Hertfordshire, Hatfield, AL10 9AB UK; 4 Precision Fuel and Hydration, Old Saw Mills, A35 Lyndhurst Road, Christchurch, Dorset, BH23 7DX UK

**Keywords:** Hydration, Sodium, Thermoregulation, Endurance, Electrolytes, Performance

## Abstract

**Purpose:**

Hypohydration of ≥ 2% body mass loss (BML) can impair cycling performance in the heat; however, it remains unclear whether this is primarily due to the body water deficit itself or the concomitant, typically observed increase in serum sodium concentration (S_[Na_^+^_]_). To disentangle these effects, we examined the performance effects of 60% (60FR) vs. 80% (80FR) fluid replacement, designed to achieve > 2% vs < 2% BML, with personalised sodium prescription targeting condition-matched S_[Na_^+^_]_.

**Methods:**

Eleven male cyclists (35 ± 6 years, VȮ_2max_ 58 ± 5 ml·kg^−1^·min^−1^) completed two trials, consisting of 180 min cycling (90% LT_1_, 32 °C, 50% humidity), followed by a work-matched time-trial (TT). Participants received either 60% or 80% of projected fluid losses, along with sodium supplementation aiming to maintain S_[Na_^+^_]_ where dilution of S_[Na_^+^_]_ was predicted. Outcomes included BML, S_[Na_^+^_]_, plasma volume change (∆PV), rectal (T_re_) and skin (T_sk_) temperatures, whole-body sweat rate (WBSR), and TT performance.

**Results:**

BML was greater in 60FR at all time-points (P < 0.01), whereas S_[[Na_^+^_]_ remained stable across both trials (P = 0.07). In 80FR, ∆PV was smaller during the preload, T_re_ was lower from 120 min onwards and T_sk_ and WBSR were higher during the TT compared to 60FR (all P < 0.05). TT completion time was 8 ± 10% faster in 80FR (1793 ± 174 vs 1968 ± 246 s, P = 0.02).

**Conclusion:**

Fluid replacement resulting in BML > 2% impairs thermoregulation and cycling performance in the heat, compared to maintaining BML < 2%, despite no differences in S_[Na_^+^_]_.

## Introduction

In prolonged endurance exercise, fluid and electrolyte balance can impact upon both performance and safety. Hypohydration leading to body mass loss (BML) ≥ 2% reduces endurance performance (Cheuvront et al. 2003; Sawka et al. 2007; James et al. 2019), particularly in hot conditions (Kenefick et al. 2010). Blinded hypohydration studies demonstrate that performance impairment can occur even when participants are unaware of their hydration status (James et al. 2017; Funnell et al. 2019). Hypovolaemia appears to be a key physiological mechanism underpinning these ergolytic effects (James et al. 2019), as reductions in plasma volume (PV) increase cardiovascular and thermal strain (Montain et al. 1998; Roy et al. 2000), and may compromise skeletal muscle perfusion (González-Alonso et al. 1998). In the presence of heat stress, hypovolaemia can also reduce skin blood flow (SkBF) (Fortney et al. 1981; Fortney and Vroman 1985), potentially impairing sweat secretion (Sawka et al. 1985) and increasing body heat storage. These effects of hypohydration are typically accompanied by elevations in serum osmolality (S_osm_) and serum sodium concentration (S_[Na_^+^_]_) (Montain and Coyle 1992), as sweat is hypotonic (Adrogué and Madias 2000; Sawka et al. 2015). Hyperosmolality may further impair thermoregulation by increasing the body temperature threshold at which sweating is initiated (Fortney et al. 1984; Lynn et al. 2012).

However, mathematical models (Kurtz and Nguyen 2003; Montain 2006; McCubbin 2022) predict that during prolonged exercise (> 3 h), particularly when fluid ingestion rates are high (≥ ~ 1 L∙h^−1^), S_[Na_^+^_]_ may remain stable or even decrease from baseline despite reduced total body water (TBW). Consistent with these predictions, and despite ongoing debate regarding the contribution of osmotically inactive sodium stores to S_[Na_^+^_]_ (McCubbin 2021), hypovolaemic hyponatraemia has been reported in ultra-endurance athletes (Hew-Butler et al. 2017; Johnson et al. 2023).

Given that high fluid ingestion rates can theoretically prevent S_[Na_^+^_]_ from rising despite hypohydration, it may be possible to isolate the effects of TBW content on performance and physiological responses independent of changes in S_[Na_^+^_]_. To date, this separation of TBW and S_[Na_^+^_]_ has not been directly examined. Despite BML being the standard metric used within hydration guidelines (Sawka et al. 2007), sweat rate varies substantially between individuals, and therefore the fluid volume required to achieve a given BML differs markedly (Baker 2017; McCubbin 2022). However, if the specific goal is to maintain a stable S_[Na_^+^_]_, as is required when mechanistically isolating the effects of TBW from those of S_[Na_^+^_]_, then prescribing fluid intake as a percentage of sweat losses is likely more appropriate than targeting a particular BML. The percentage fluid replacement approach avoids substantial increases in S_[Na_^+^_]_ in individuals who require less fluid to maintain a given BML (Kurtz and Nguyen 2003; McCubbin 2022), enabling tighter control of S_[Na_^+^_]_. For example, providing a fluid volume predicted to dilute S_[Na_^+^_]_ (~ 80% replacement) in one condition, and a lower fluid volume predicted to induce minimal change in S_[Na_^+^_]_ (~ 60% replacement) in another, while administering personalised, pre-calculated sodium (Na^+^) intake whenever dilution of S_[Na_^+^_]_ is anticipated (McCubbin 2022), would permit comparison of differing TBW levels with matched S_[Na_^+^_]_.

The primary aim of this study was to compare cycling performance, physiological, and perceptual responses in the heat between two fluid replacement strategies (60% of losses; 60FR vs 80% of losses; 80FR), which would elicit different BML/TBW losses, with matched S_[Na_^+^_]_ between trials. Stability of S_[Na_^+^_]_ between trials was achieved using personalised Na^+^ ingestion where S_[Na_^+^_]_ dilution was predicted, guided by mathematical modelling (McCubbin 2022).

## Methods

### Participant characteristics and inclusion criteria

An A priori power calculation (G*Power 3.1) was performed (0.05 alpha, 90% power) using performance data from a previous hydration study (Logan-Sprenger et al. 2015), which included a time-trial (TT) with a similar work target (6 kJ∙kg BM^−1^ vs 6.1 ± 0.7 kJ∙kg BM^−1^ in the present study). This gave a required sample size of eleven.

**Fig. 1:**
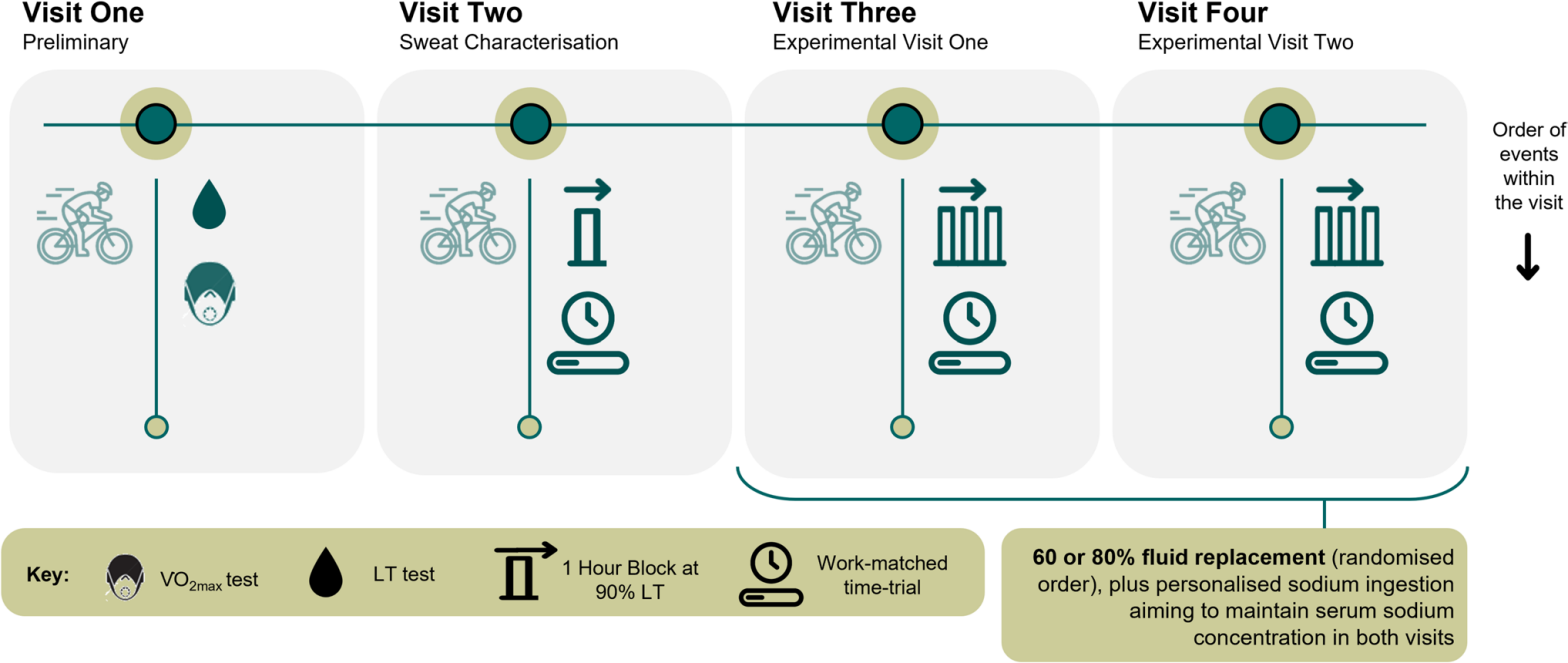
Overview of visits and order of main events during each visit. The time-trial task in visit two was completed as a familiarisation to minimise any learning effect ahead of the experimental visits

Eleven non-heat acclimated, Tier 2 or 3 (trained or highly trained; McKay et al. 2022) male cyclists completed the study. Mean ± SD participant characteristics were: age 35.0 ± 6.4 years, BM 76.0 ± 6.6 kg, body fat 15.3 ± 4.0%, stature 180.4 ± 5.5 cm and V̇O_2max_ 58 ± 5 ml·kg^−1^·min^−1^. Inclusion required ≥ 6 h of cycling training per week for ≥ 3 yrs. Women were not included in this study due to the potential influence of menstrual cycle–related hormonal fluctuations on thermoregulation (Baker et al. 2020) and fluid/electrolyte balance (Rodriguez‐Giustiniani et al. 2022), which could have introduced additional variability. Exclusion criteria included a maximum oxygen consumption (V̇O_2max_) < 45 ml∙kg^−1^∙min^−1^, smoking, blood pressure > 140/90 mmHg and conditions affecting sweat gland/renal function. Prior to commencing data collection, ethical approval was obtained from the University of Bedfordshire’s Institute for Sport and Physical Activity Research Ethics Committee (Approval Number: 2023ISPAR005). Written informed consent was obtained, and data were collected in-line with the Declaration of Helsinki. All participants completed four visits (Fig. 1). Experimental trials were completed seven days apart, at the same time of day (standardised within participant), in a double-blind, computer-generated, block balanced randomised order.

### Control of diet and exercise

Over the 48-h period preceding each laboratory trial, participants replicated their dietary intake and physical activity and recorded these in logs, which were collected by the lead researcher. Participants were provided with a standardised breakfast to prepare and consume at home before arriving at the laboratory. The breakfast consisted of two 38.5 g sachets of porridge oats (Quaker Oat So Simple Big Bowl, Quaker Oats, Chicago, IL, USA) made with 490 mL of milk, plus an additional 500 mL of bottled water. The approximate overall nutritional composition of the standardised breakfast was: energy 698 kcal, CHO 91 g, protein 30 g, fat 17 g, Na^+^ 204 mg, total fluid 990 mL. Participants refrained from alcohol for 24 h and from vigorous exercise for 48 h before each visit. No caffeine was consumed in the 24 h prior to trials, due to its ability to alter thermoregulatory responses (Hunt et al. 2021).

### Preliminary visit

Blood pressure (Omron Evolv, HEM-7600 T-E, MRON Healthcare Europe BV) and stature (Harpenden, HAR-98.602, Holtain) were measured. BM and body fat were assessed using bioelectrical impedance analysis scales (SECA mBCA515, SECA Group, Hamburg, Germany).

Participants then completed a two-section incremental exercise protocol on a cycle ergometer (Lode Excalibur Sport, Netherlands) in temperate conditions (temperature 21.4 ± 1.2 °C, humidity 50.7 ± 9.4%) as previously described (Newell et al. 2015). Capillary blood lactate (Biosen C-Line, EKF Diagnostics, Cardiff, UK) was sampled during the final 30 s of each 4 min stage. The first lactate threshold (LT_1_) was determined as the first rise in blood [lactate] above baseline, judged through visual inspection (Davis et al. 2007). LT_1_ was independently assessed by two researchers and consensus reached to confirm LT_1_ (195 ± 20 W). After a 10 min rest, section two determined V̇O_2max_ (58 ± 5 ml·kg^−1^·min^−1^) and peak power output (PPO; 391 ± 37 W). Expired gas analysis occurred throughout section two (Cortex Metalyzer, Leipzig, Germany), with the highest V̇O_2_ averaged over 30 s taken as V̇O_2max_ (Newell et al. 2015).

### Sweat characterisation visit

On arrival, participants emptied their bladder and urine osmolality (U_osm_) was measured (Atago-Vitech-Scientific, Pocket-PAL-OSMO, HaB-Direct, Southam). Pre-exercise euhydration was defined as U_osm_ ≤ 600 mOsm∙kg H_2_O^−1^ (Aldous et al. 2016); if exceeded, 250 mL water was provided and U_osm_ was re-checked when participants were able to produce another urine sample. Total body water (TBW) was assessed via bioelectrical impedance analysis (SECA mBCA515) and corrected using deuterium-dilution regression equations specific to trained male endurance athletes (McCubbin & Nguo, unpublished).

Participants cycled on a Lode ergometer at 90% LT_1_ (176 ± 18 W; 45 ± 5% of PPO) for one hour, in an environmental chamber (TISS Peak Performance Chamber, TIS Services, Hampshire, UK; temperature 32.0 ± 0.4 °C**,** humidity 54.1 ± 4.8%, facing wind speed 1.4—1.6 m∙s^−1^). No fluid was ingested by participants. Expired gases were sampled for 3 min at 15 and 45 min. At 30 min, participants stopped cycling for ~ 3 min, so that 5 × 7 cm absorbent patches (Tegaderm + Pad, 3 M Health Care, Minnesota, USA) could be applied to the posterior mid-forearm and the anterior mid-thigh on the right and left side of the body. Patch sites were shaved before exercise and rinsed with deionised water and thoroughly dried prior to application of each patch. At 60 min, patches were removed with tweezers that had been pre-rinsed in deionised water and dried, before being placed into a Salivette® tube (Sarstedt, Nümbrecht, Germany) and centrifuged at 4000 rpm for 10 min, to extract sweat. Right-sided patches were used to determine whole-body sweat electrolytes, except where a right-sided patch was poorly adhered, in which case the left was used.

Post exercise urine (0.238 ± 0.093 L) was collected and urine [Na^+^] (27.54 ± 18.45 mmol∙L^−1^) and [K^+^] (34.53 ± 13.74 mmol∙L^−1^) determined, as detailed below**.** Whole-body sweat loss (WBSL) was determined from ∆BM, with mass of ingested carbohydrate chews (PF 30 Chews Original, Precision Fuel and Hydration, Christchurch, UK), mass loss due to substrate oxidation (Jeukendrup and Wallis 2005; Maughan et al. 2007), respiratory water loss (Mitchell et al. 1972) and metabolic water production (Maughan et al. 2007) all accounted for.

Following the 1 h bout, participants completed a familiarisation TT, identical to that performed in subsequent experimental visits, to minimise any learning effect.

### Experimental visits

Pre-exercise U_osm_ analysis and TBW measurement procedures matched the sweat characterisation (SC) visit. A peripheral venous cannula (Verastus-WP, Terumo, Belgium) was inserted into an antecubital vein, and a single-lumen extension line was attached (Octopus 1, Vygon, France).

### Submaximal preload exercise bout

**Fig. 2:**
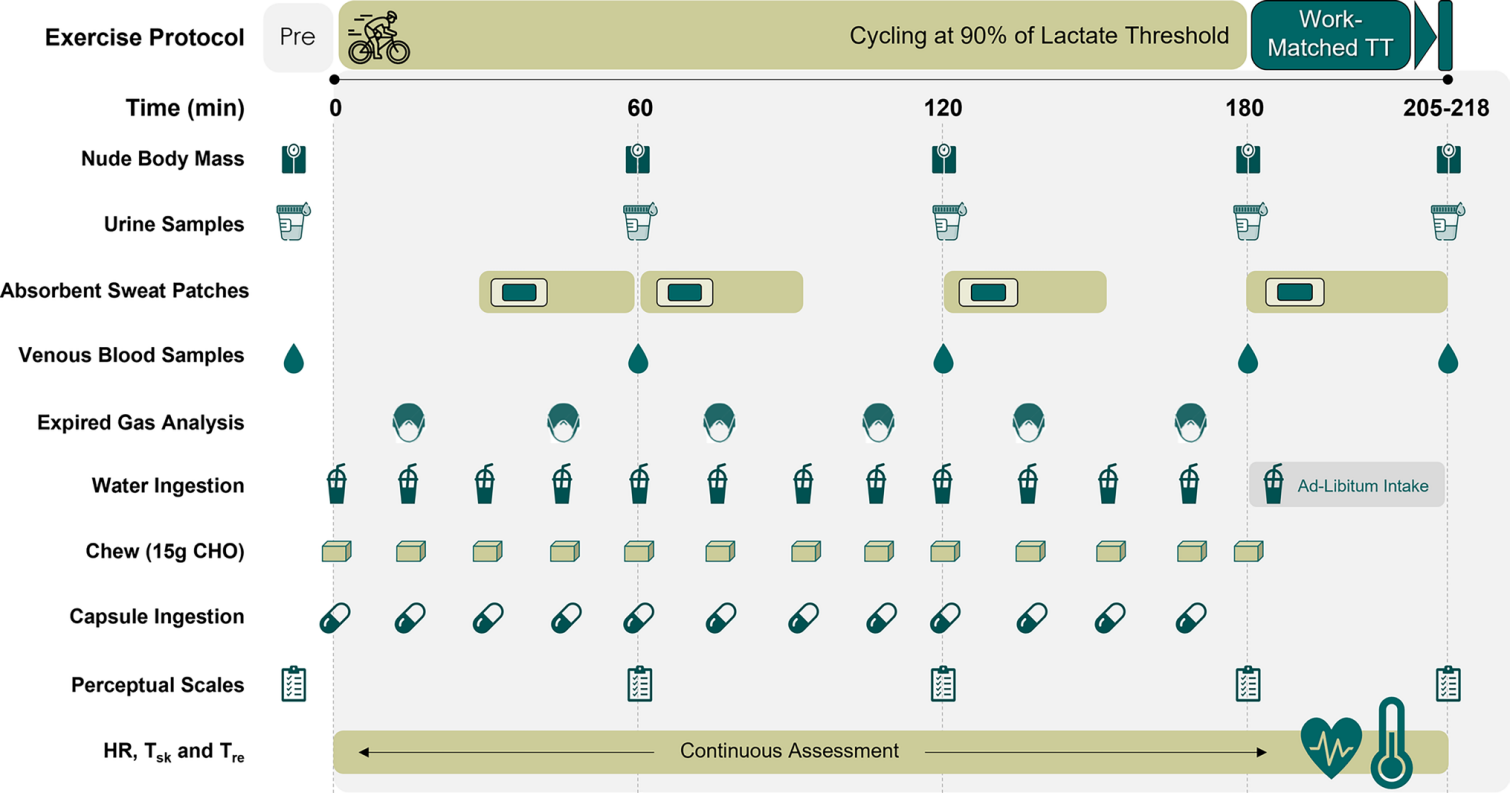
Experimental trial timeline indicating the frequency of measurements and nutritional strategy. Capsules ingested either contained sodium chloride or were empty depending upon estimated serum sodium concentration calculations (McCubbin 2022). CHO = carbohydrate, HR = heart rate, T_sk_ = skin temperature and T_re_ = rectal temperature

Participants cycled for 3 h at 90% LT_1_, split into three 1-h blocks to allow brief stops for urine and BM measures. Cycling exercise occurred in an environmental chamber, with no significant differences in ambient temperature (80FR 31.9 ± 0.4 °C; 60FR 31.9 ± 0.1 °C, P = 0.286) or humidity (80FR 52.6 ± 1.9%; 60FR 51.6 ± 2.2%, P = 0.288) between trials. Patches were applied for 30 min during each 1-h block: from 30—60 min in hour 1 and 0—30 min in hour 2 and 3, as sweating would have reached steady-state (Baker 2017). All patch application, removal and site preparation occurred as in the SC visit. Measurement frequency and feeding strategy are shown in Fig. 2.

### Hydration and feeding strategy

Fluid and Na^+^ ingestion strategies were based on projected fluid, Na^+^ and potassium losses through both sweat and urine in the SC visit. Fluid and Na^+^ were ingested in 12 equal doses across the three-hour preload (Table 1). The selected fluid replacement levels (60% and 80%) were informed by WBSRs measured during pilot testing. Calculations indicated that these levels would, on average, cause BML above and below 2% respectively, thereby allowing trials to straddle the commonly cited performance threshold. In both trials, personalised Na^+^ supplementation was calculated using established mathematical models (Kurtz and Nguyen 2003; McCubbin 2022). Sodium was provided whenever modelling predicted that ingestion of water alone would dilute S_[Na_^+^_]_, enabling comparable S_[Na_^+^_]_ between trials despite differing TBW losses. Based on pilot data and the known physiological ranges of sweat [Na^+^] in endurance athletes (Baker 2017; Barnes et al. 2019), these fluid replacement levels were expected to generate different Na^+^ requirements between trials. Specifically, 80FR was anticipated to require exogenous Na^+^ supplementation in all participants, whereas 60FR was expected to produce minimal changes in S_[Na_^+^_]_ in most participants, with additional Na^+^ intake required to maintain S_[Na_^+^_]_ at baseline only in participants with high sweat [Na^+^] (> ~ 65 mmol∙L^−1^). The quantity of Na^+^ required to maintain S_[Na_^+^_]_ was calculated using the following equation (McCubbin 2022):

$$\begin{gathered} Na^{ + } {\mathrm{intake}} = \hfill \\ \quad \frac{{\left[ {TBW_{{post}} \times \left( {S_{{\left[ {Na^{ + } } \right]post}} + 23.8} \right)} \right] - \left[ {TBW_{{pre}} \times \left( {S_{{\left[ {Na^{ + } } \right]pre}} + 23.8} \right)} \right]}}{{1.03}} \hfill \\ \quad + (Na^{ + } {\text{ }}loss + K^{ + } loss - K^{ + } {\text{ }}\mathrm{int} ake) \hfill \\ \end{gathered}$$

where _pre_ and _post_ refer to pre- and post-exercise, Na^+^ and K^+^ loss / intake are in mmol, TBW is in L, S_[Na_^+^_]_ is serum sodium concentration in mmol∙L^−1^ and Na^+^ / K^+^ loss includes losses in sweat and urine. The original equation (Kurtz and Nguyen 2003; McCubbin 2022) used plasma [Na^+^], but since S_[Na_^+^_]_ was measured in laboratory validations of the Kurtz-Nguyen equation (Baker et al. 2008), it has been used here instead.

**Table 1:** Fluid intake (L), sodium intake (mmol) and relative sodium intake per litre of fluid consumed (mmol∙L^−1^) intakes during the preload in the two experimental trials

	80% fluid replacement	60% fluid replacement	P-value (60% vs 80%)
Fluid ingestion rate (L∙h^−1^)	1.125 ± 0.269	0.844 ± 0.202	< 0.001
Sodium ingestion rate (mmol∙h^−1^)	29.66 ± 26.25 (n = 11 ingested sodium)	4.34 ± 10.52 (n = 3 ingested sodium)	0.003
Relative sodium ingestion per L of fluid consumed (mmol∙L^−1^)	24.48 ± 20.50	4.81 ± 11.65	< 0.001

^Values are shown as mean ± SD^

Na^+^ was provided to participants via NaCl in 12 opaque hydroxypropyl methylcellulose capsules (Size 1, Length ~ 19.4 mm, 0.50 mL volume; YourSupplements, Stoke-on-Trent, UK) over 3 h. If modelling predicted that individuals did not require Na^+^ to maintain S_[Na_^+^_]_ during a trial, then empty capsules were provided. Carbohydrate chews were provided along with each water and capsule, to provide sufficient fuel for the 180 min exercise bout (60 g∙h^−1^ carbohydrate, 195 g total; PF 30 Chews Original, Precision Fuel and Hydration, Christchurch, UK). Immediately prior to the TT, participants consumed a final 15 g carbohydrate chew. A total of 3 L of water was available to consume ad-libitum during the TT, but no capsules or carbohydrate were consumed. All drinks consumed during the preload and the TT were stored in the fridge up until the point of consumption (beverage temperature ~ 6 – 7 °C).

### Measures

Rectal temperature (T_re_) was measured using a single-use rectal thermistor (Henleys, 400H, Henleys), inserted 10 cm past the anal sphincter. Skin temperature (T_sk_) was measured using iButton^®^ wireless thermistors (Maxim Integrated Products, Inc., Sunnyvale, California, USA), placed at four sites on the right side of the body (gastrocnemius, triceps brachii, rectus femoris, and pectoralis major), to calculate mean T_sk_ (Ramanathan 1964). T_sk_, T_re_ and HR (Polar H10, Polar Electro Oy, Kempele, Finland) values were recorded every 30 min during the preload and every 20% of work target completed during the TT. Thirst sensation (Engell et al. 1987), thermal comfort (Attia et al. 1980) and rating of perceived exertion (RPE; Borg 1982) were measured each hour, and post TT.

Venous blood samples were drawn hourly and post TT. A 5 mL blood sample was collected into a serum-separating tube (SST^™^ II Advance; BD Vacutainer^®^, Becton, Dickinson and Company, Franklin Lakes, NJ, USA) and left at room temperature for 30 min. A 6 mL sample was collected into an EDTA-treated tube (K₂ EDTA 10.8 mg; BD Vacutainer^®^, Becton, Dickinson and Company, Franklin Lakes, NJ, USA) and placed on ice for 30 min. After each blood draw, the line was flushed with 1 mL of sterile saline solution (0.9% NaCl; PosiFlush^™^, BD, Franklin Lakes, New Jersey, USA) to keep it patent. At subsequent draws, 2 mL was discarded before sampling to avoid dilution. Baseline blood samples were drawn immediately after cannula insertion, whilst participants remained supine. All other blood draws occurred whilst participants were cycling.

Participants voided at the end of each hour and post TT, prior to drying sweat off the skin and measuring nude BM (1 decimal place; TANITA Corporation, Tokyo, Japan). Not all participants were able to urinate at every timepoint: one was unable to urinate at 120 min, two at 180 min and one post TT.

Expired gas was measured for 3 min at 15 and 45 min through each hour, to estimate non-sweat contributions to mass balance, as done during the SC visit. There were no expired gas measures during the TT, so WBSL values were corrected for non-sweat contributions to mass balance as follows:

$$\text{WBSL }= \Delta \text{BM }\times 0.20\mathrm{g}\cdot \mathrm{kcal}{}^{-1}$$

where 0.20 g∙kcal^−1^ represents estimated non-sweat mass loss through respiratory water loss and gas exchange (Cheuvront and Montain 2017; Cheuvront and Kenefick 2017). TT energy expenditure (kcal) was estimated using published equations (Van Hooren et al. 2023). WBSL during the TT was divided by exercise duration to give whole-body sweat rate (WBSR).

### Time-trial

The TT consisted of completing a specific work target in the shortest possible time, with no verbal motivation, other than to notify participants of completion of 50%, 75% and 90%. Work target was equivalent to cycling at 65% PPO for 30 min. Work targets and linear factors were calculated and entered into Lode Ergometry Manager as previously explained (Newell et al. 2015).

Trials were terminated if T_re_ exceeded 39.7 °C, which occurred for one participant during the TT in 60FR trial and for another participant during the TT in 80FR. A third participant was unable to complete the entire work target in 60FR due to a hamstring cramp. In all three of these cases, the TT was terminated when > 95% (96.3 ± 1.2%) of work target had been completed. The work targets for those three participants were therefore normalised to the work completed at the point of termination in the trial that was cut short, to enable fair comparisons between trials.

### Sample analysis

Whole venous blood samples from hourly draws were analysed immediately for haemoglobin concentration (HemoCue Hb 201 + ; HemoCue AB, Ängelholm, Sweden) and haematocrit (Microcentrifugation; Hawksley Microhematocrit Centrifuge, Hawksley, Worthing, UK), to calculate change in plasma volume from baseline (∆PV) (Dill and Costill 1974). S_[Na_^+^_]_, serum potassium concentration (S_[K_^+^_]_) and serum chloride concentration (S_[Cl_^−^_]_) were measured immediately after centrifugation by ion selective electrodes (Prolyte Electrolyte Analyzer, Diamond Diagnostics, Hungary). Two participant’s blood data are excluded from both trials, due to complications obtaining and analysing samples (N = 9 for blood measures).

Urine samples were first assessed for volume and U_osm_. After centrifugation, urine samples were diluted 1:10 with urine diluent (Diamond Diagnostics, Hungary), before urine [Na^+^], [K^+^] and [Cl^−^] were determined through ion selective electrode analysis (Prolyte Electrolyte Analyzer, Diamond Diagnostics, Hungary).

Local sweat rate (LSR) was measured by dividing the change in mass of the Tegaderm by the pad surface area and the length of application (Smith and Havenith 2011). Sweat [Na^+^] and [K^+^] were assessed using ion selective electrodes (LAQUATwin, Horiba, Kyoto, Japan). Local sweat [Na^+^] and [K^+^] values were converted to estimated whole-body values using regression equations (Baker et al. 2009).

### Statistical analysis

Data are presented as mean ± SD, statistical analysis was completed using IBM SPSS Statistics (Chicago, IL) and P < 0.05 was taken to be statistically significant. For variables with multiple timepoints, linear mixed models were used to determine condition, time and interaction effects. Linear mixed models allow for missing data points, flexible modelling of covariate structures and account for variation between participants (Vandenbogaerde and Hopkins 2010; Aldous et al. 2016; West et al. 2022). Sidak-adjusted post-hoc tests were used to determine differences between condition and/or time when there was a significant interaction. In each instance, the model with the lowest Hurvich and Tsai’s criterion (AICC) was taken as the most suitable, in line with the principle of parsimony. Residuals were assessed for normality through inspection of Q-Q plots. For variables with one timepoint, a paired t-test was used, with Q-Q plots being checked to confirm normality. Normality was violated for comparisons of ambient temperature, so a Wilcoxon signed-rank test was used. Where differences in a variable between trials are quantified, they are presented as mean difference with associated 95% confidence limits.

## Results

### Baseline measures

Baseline U_osm_ (80 FR 238 ± 157 mOsm∙kg H_2_O^−1^; 60FR 239 ± 160 mOsm∙kg H_2_O^−1^ P = 0.971), BM (80FR 76.0 ± 6.8 kg; 60FR 75.6 ± 6.7 kg, P = 0.194), TBW (80FR 45.21 ± 3.11 L; 60FR 44.93 ± 3.27 L, P = 0.074), and thirst (80FR 3 ± 1; 60FR 4 ± 1, P = 0.064) were not significantly different between trials. There was a significant difference in baseline S_[Na_^+^_]_ between trials (80FR 138.97 ± 1.59 mmol∙L^−1^; 60FR 138.21 ± 1.32 mmol∙L^−1^, P = 0.024) and therefore baseline S_[Na_^+^_]_ was treated as a covariate in any future trial* time LMM analysis involving S_[Na_^+^_]_.

### Hydration status

**Fig. 3:**
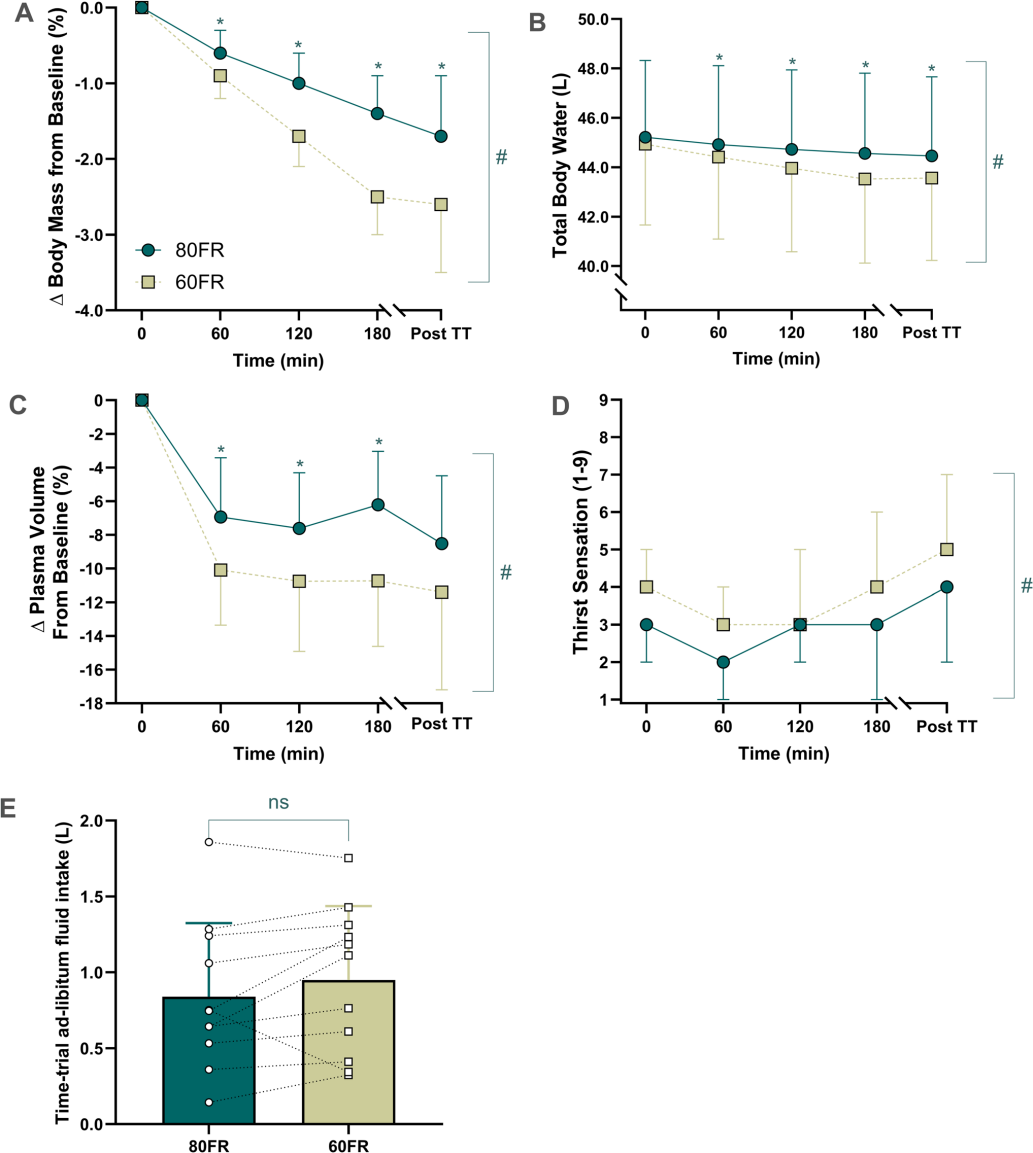
Body mass change from baseline (**A**), total body water (**B**), plasma volume change from baseline (**C**) and thirst sensation (**D**) during the 180 min preload exercise bout and post time-trial, and ad-libitum fluid ingestion during the time-trial (**E**) for the eighty (80FR) and sixty (60FR) percent fluid replacement conditions. * = significant (P < 0.05) difference between trials at timepoint, # = significant (P < 0.05) effect of trial, ns = no significant difference (P > 0.05). The sample size for plasma volume measures was n = 9, but there was a complete dataset (n = 11) for all other measures presented in the figure

∆BM, TBW and ∆PV all demonstrated significant time, trial and interaction effects (P ≤ 0.039). When compared to 80FR, 60FR elicited a greater reduction in BM from baseline at all time-points apart from baseline (P ≤ 0.034; Fig. 3a), notably at 180 min (2.5 ± 0.5 vs 1.4 ± 0.5%) and post-TT (2.6 ± 0.9 vs 1.7 ± 0.8%). TBW was also higher in 80FR than in 60FR from 60 min onwards (P ≤ 0.01; Fig. 3b) and was 1.0 L higher post-preload (95% CI: + 0.7 to + 1.4 L). ∆PV was different between trials at all timepoints during the preload (P ≤ 0.033), but not post-TT (P = 0.076). Post-preload, PV reductions were attenuated by 5% in 80FR (95% CI: -2 to -8%). For thirst, there were effects of time and trial (P ≤ 0.013), with thirst higher in 60FR across all timepoints, but no interaction effect (P = 0.418; Fig. 3d).

**Fig. 4:**
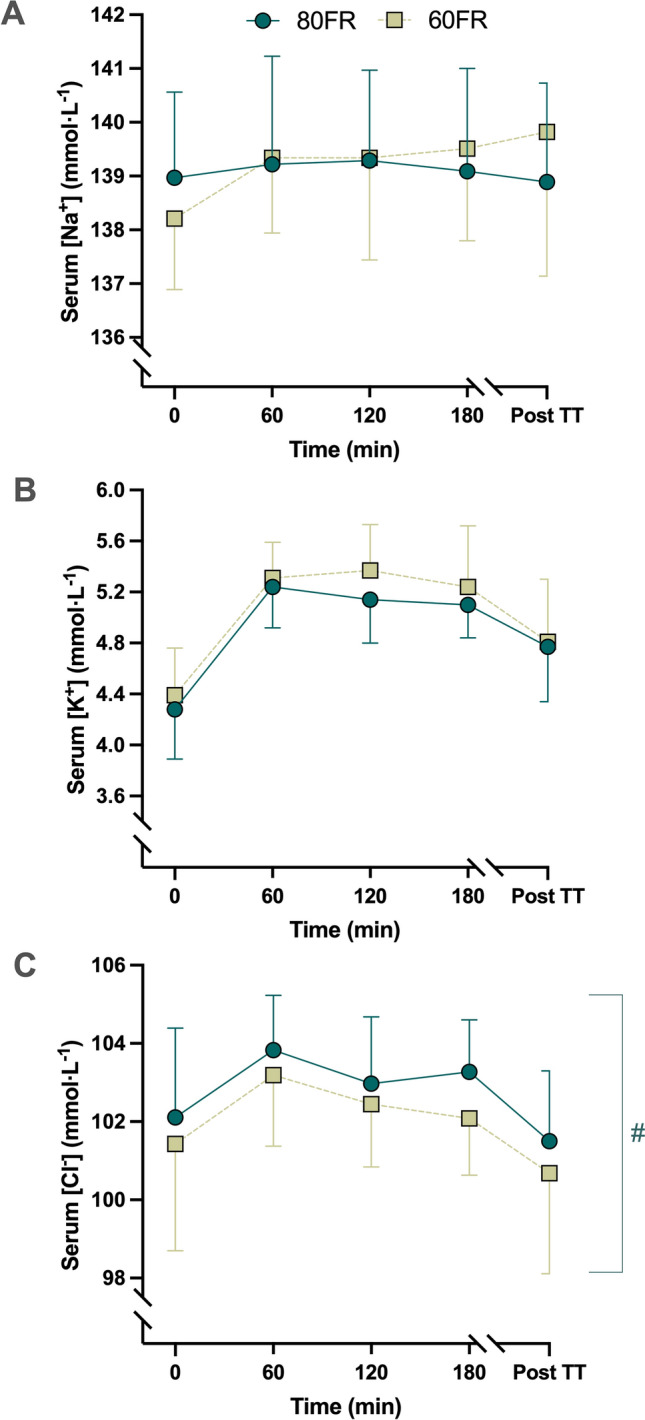
Serum concentrations of sodium (**A**), potassium (**B**) and chloride (**C**) during the 180 min preload exercise bout and post time-trial for the eighty (80FR) and sixty (60FR) percent fluid replacement conditions. # = significant effect of trial. n = 9 for all serum electrolyte data (difficulties obtaining/analysing blood samples for two participants)

Ad-libitum fluid ingestion during the TT was not significantly different between trials (80FR 0.839 ± 0.485 L, 60FR 0.949 ± 0.487 L, P = 0.167, Fig. 3e). Ad-libitum fluid ingestion during the TT was sufficient to maintain post-preload hydration levels, since ∆BM and TBW were not significantly different between 180 min and post TT in either trial (P ≥ 0.544; Fig. 4a and Fig. 4b).

### Serum electrolyte concentrations

S_[Na_^+^_]_ remained stable over time (P = 0.566), did not differ between trials (P = 0.303) and did not have a significant interaction effect (P = 0.067; Fig. 4a). To achieve this stability of S_[Na_^+^_]_, all participants required exogenous Na^+^ in 80FR (29.66 ± 26.25 mmol∙h^−1^), whereas only 3 participants required Na^+^ in 60FR (4.34 ± 10.52 mmol∙h^−1^). S_[K_^+^_]_ increased significantly from baseline in both trials (P < 0.001) but showed similar responses between trials (P = 0.086; Fig. 4b). For S_[Cl_^−^_]_, there were effects of trial (P = 0.002) and time (P < 0.001), but no interaction (P = 0.890; Fig. 4c).

### Thermoregulatory variables

**Fig. 5:**
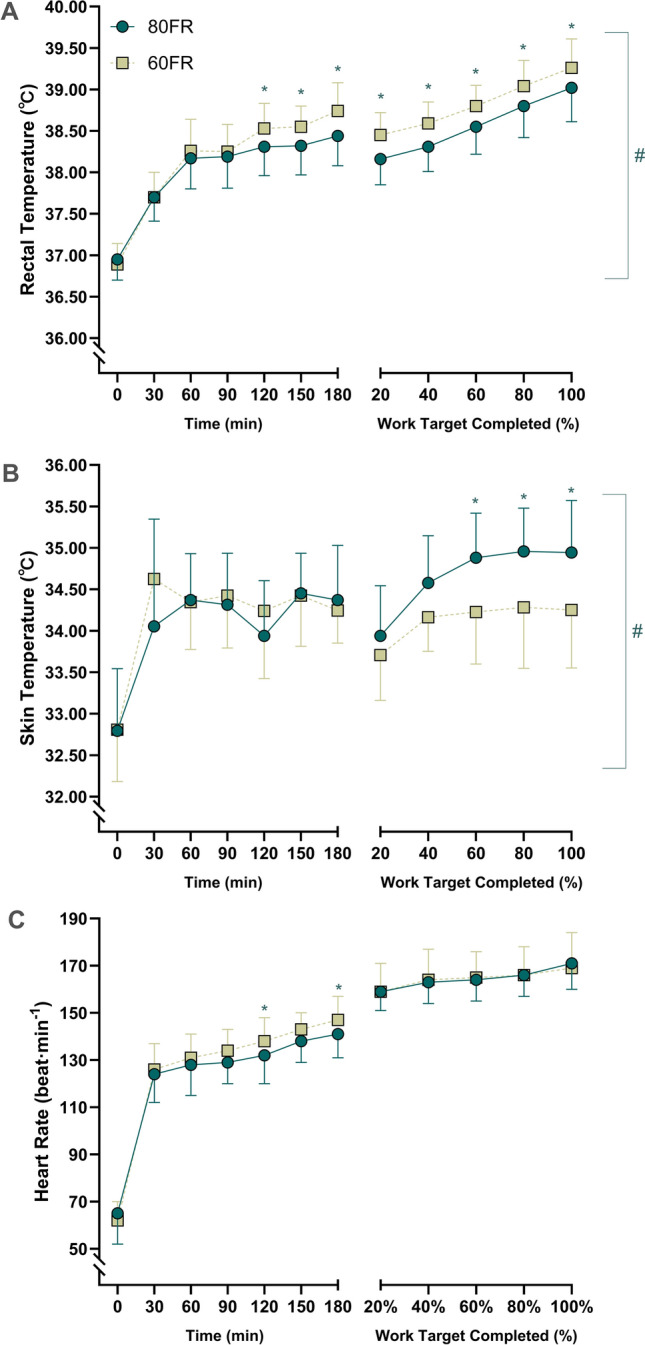
Rectal temperature (**A**), skin temperature (**B**) and heart rate (**C**) every 30 min during the 180 min preload exercise bout and every 20% of work target completed during the time-trial for the eighty (80FR) and sixty (60FR) percent fluid replacement conditions. * = significant (P < 0.05) difference between trials at timepoint, # = significant (P < 0.05) effect of trial

T_re_ and T_sk_ increased markedly from baseline in both trials (time effect, both P < 0.001). However, the time-course changes in T_re_ and T_sk_ responses differed between trials (interaction effects, P ≤ 0.049). T_re_ responses diverged between trials from 120 min onwards (P ≤ 0.012), with participant’s T_re_ being 0.30 °C lower in 80FR at the end of the preload (95% CI: -0.47 to -0.13 °C). T_re_ also remained lower during the TT in 80FR compared to 60FR (P ≤ 0.007). T_sk_ did not differ between trials during the preload or the early stages of the TT (P ≥ 0.072), but was higher in 80FR at 60%, 80% and 100% of work done during the TT (P ≤ 0.021). HR increased over the exercise bout (time effect, P < 0.001); however, the magnitude of cardiovascular drift experienced differed between trials (interaction effect, P = 0.045; Fig. 5c). Specifically, when compared to 60FR, HR was lower in 80FR at 120 and 180 min during the preload (P ≤ 0.034) but was not different at any other timepoints (P ≥ 0.063).

### Urine and sweat losses

**Fig. 6:**
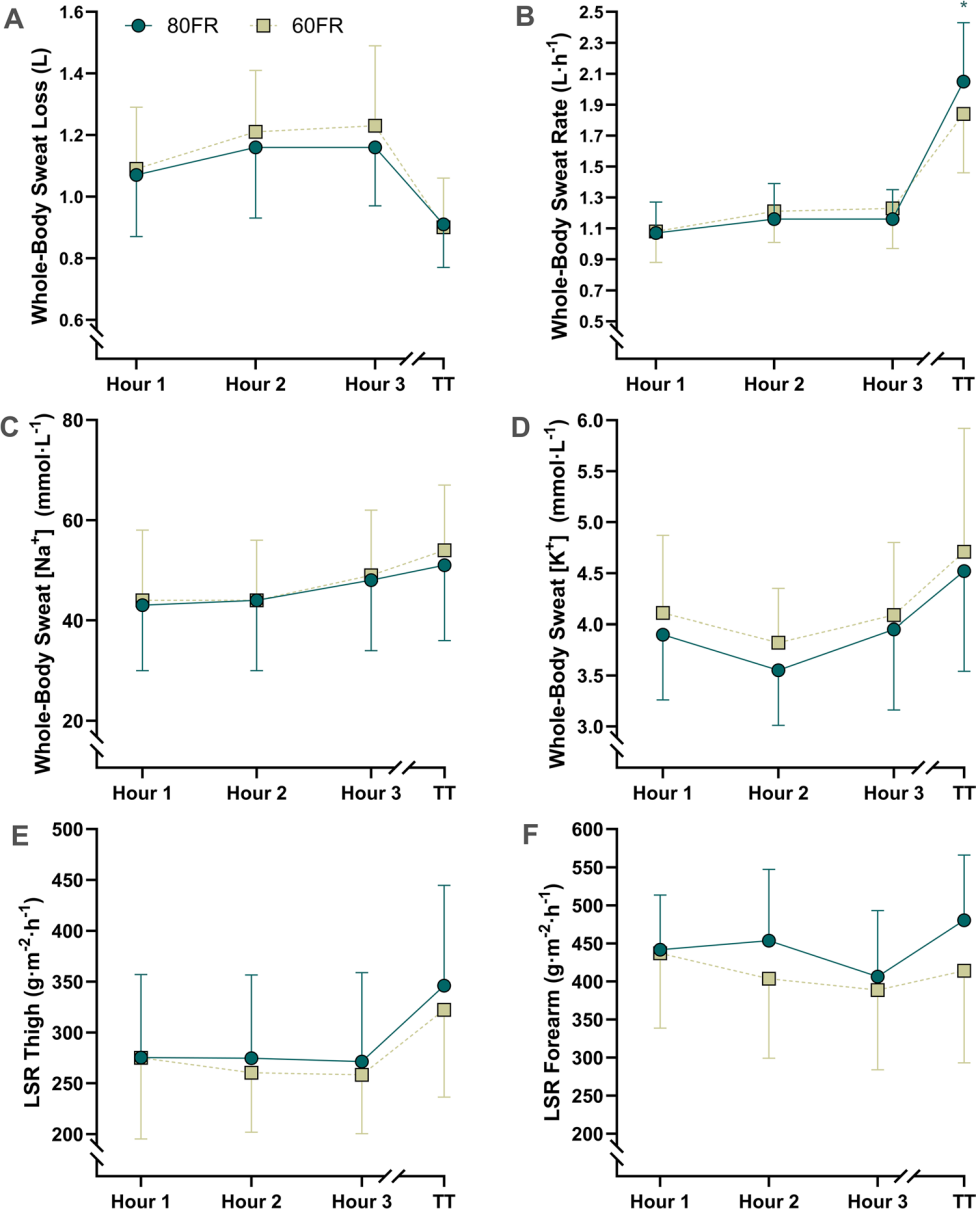
Whole-body sweat loss (**A**), whole-body sweat rate (**B**) estimated whole-body sweat sodium concentration (**C**), estimated whole-body sweat potassium concentration (**D**) and local sweat rate on the thigh (**E**) and forearm (F) during the 180 min preload exercise bout and post time-trial for the eighty (80FR) and sixty (60FR) percent fluid replacement conditions

Total urine volume produced during the steady-state and post-TT (excluding baseline) was higher in 80FR (0.609 L ± 0.332 L vs 0.413 ± 0.236 L, P = 0.011). However, total urine Na^+^ losses (80FR 18.43 ± 11.04 mmol; 60FR 11.02 ± 3.97 mmol, P = 0.055) and total urine K^+^ losses (80FR 26.21 ± 9.33 mmol; 60FR 21.85 ± 9.15 mmol, P = 0.107) did not differ between trials. Absolute WBSL values and time-corrected WBSR values are shown in Fig. 6a and b. WBSL did not differ between trials; however, an interaction effect was observed WBSR (P = 0.01; Fig. 6b), with WBSR being significantly higher in 80FR during the TT (P = 0.001), despite no differences during the preload. Sweat electrolyte concentrations ([Na^+^] and [K^+^]) also did not differ between trials (P ≥ 0.137; Fig. 6c and Fig. 6d), despite increasing over the course of the exercise bout in both trials (time effect, both P < 0.001). Thigh and forearm LSRs also did not differ between trials (P ≥ 0.181), as shown in Fig. 6e and f.

### Perceptual measures

**Fig. 7:**
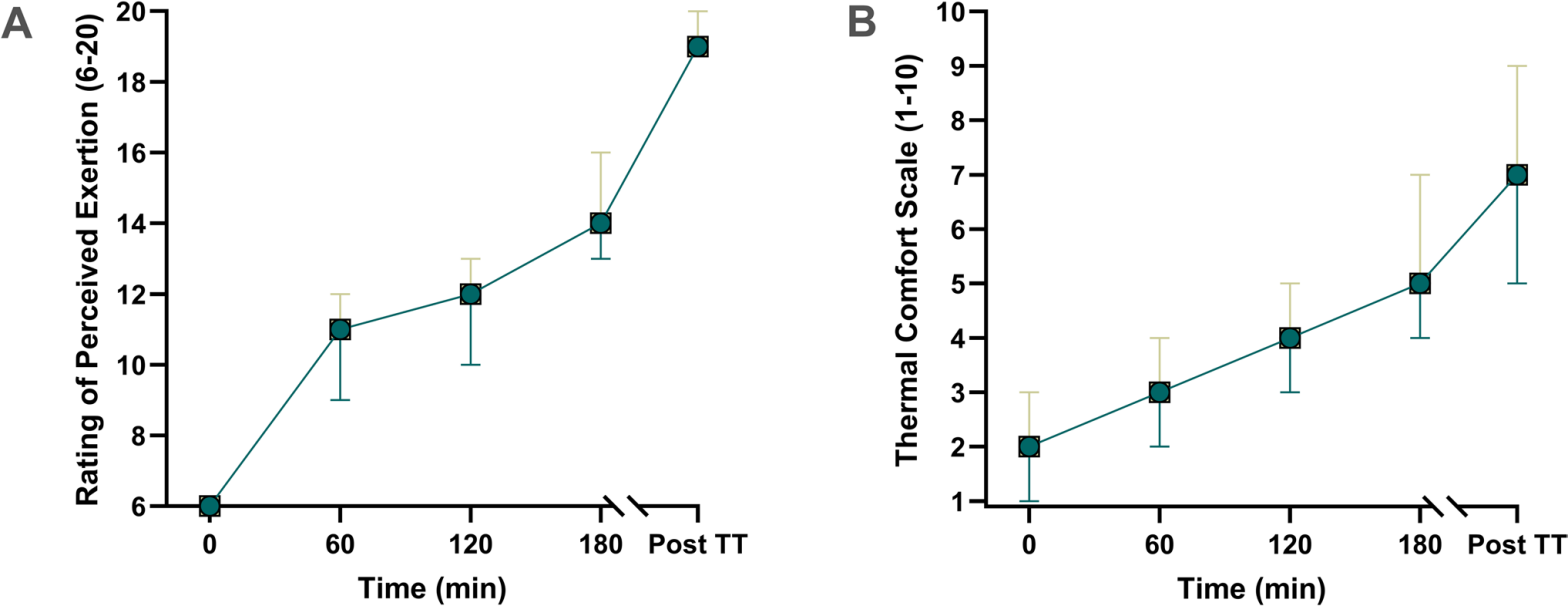
Rating of Perceived Exertion (**A**) and thermal comfort (**B**) during the 180 min preload exercise bout and post time-trial for the eighty (80FR) and sixty (60FR) percent fluid replacement conditions

Thermal comfort and RPE increased over time (both P < 0.001) but did not differ between trials (P ≥ 0.523; Fig. 7a and b).

### Time-trial performance

**Fig. 8:**
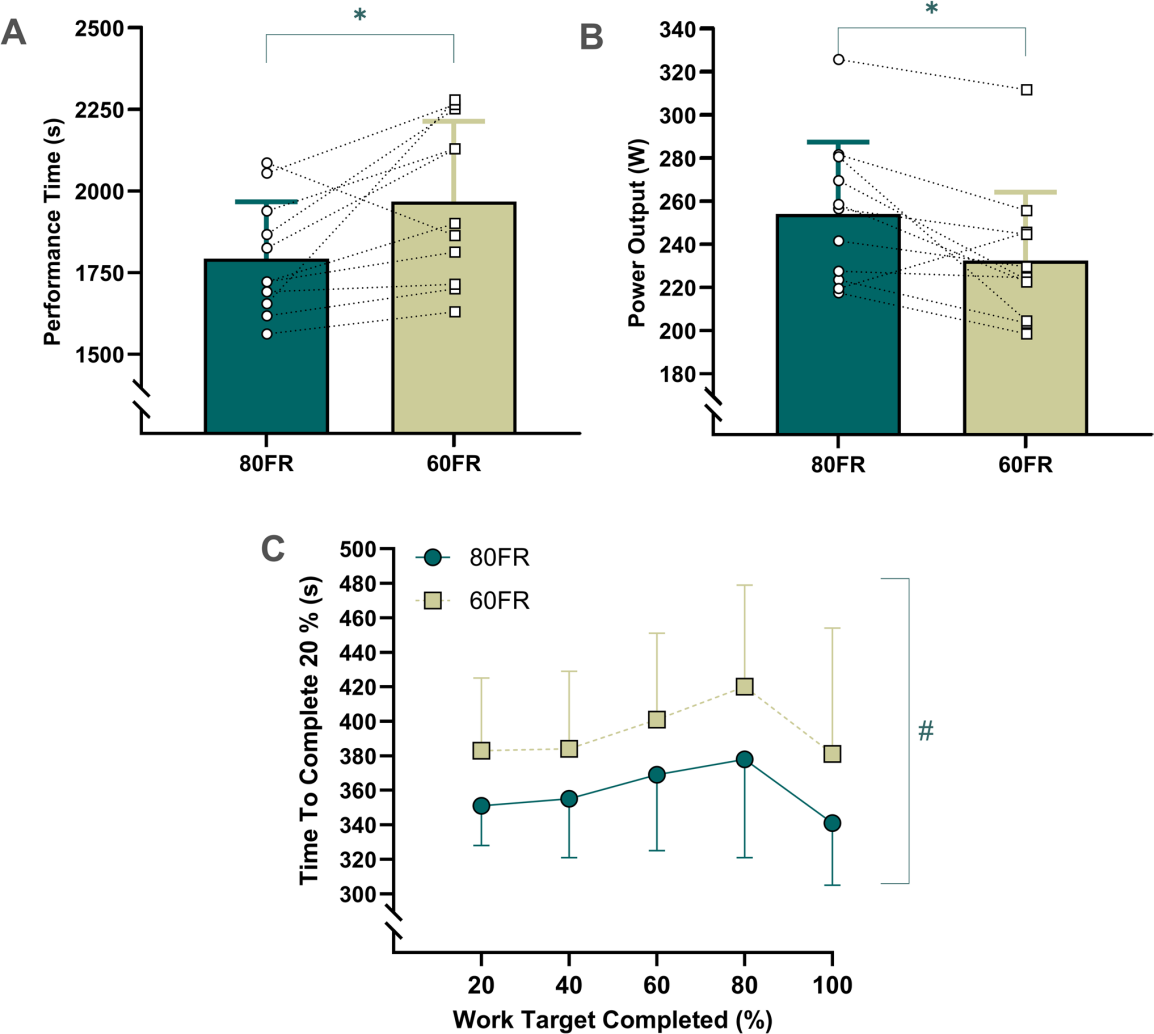
Total completion time (**A**), mean power output (**B**), time taken to complete each 20% segment (**C**) during the time-trial for the eighty (80FR) and sixty (60FR) percent fluid replacement conditions. * = significant (P < 0.05) difference between conditions, # = significant (P < 0.05) effect of trial, with faster time to complete each segment in 80FR

Completion time was 8 ± 10% faster in 80FR, with a mean improvement of 175 s in comparison to 60FR (95% CI: -30 to -321 s, P = 0.023; Fig. 8a). Average power was 22 W higher in 80FR when compared with 60FR (95% CI: + 4 to + 39 W, P = 0.019; Fig. 8b). Completion time for each 20% of work demonstrated time (P < 0.001) and trial (P = 0.012) effects, but no interaction (P = 0.660; Fig. 8c).

## Discussion

This study compared cycling performance and thermoregulatory responses between replacing 60% (60FR) versus 80% (80FR) of fluid losses during 3 h of cycling in the heat, while personalising Na⁺ ingestion to maintain S_[Na_^+^_]_ across trials. Despite matched S_[Na_^+^_]_, the greater hypohydration in 60FR impaired subsequent TT performance: completion time was 8% faster in 80FR (− 175 s, 95% CI − 321 to − 30 s, Fig. 8a) and mean power was + 22 W (95% CI + 4 to + 39 W, Fig. 8b). These findings indicate that hypohydration and the resulting hypovolaemia can hinder endurance performance in the heat independent from changes in S_[Na_^+^_]_.

Post-preload, BML was attenuated by 1.0% in 80FR (95% CI -0.7 to -1.3%, Fig. 3a), TBW was + 1.0 L higher (95% CI: + 0.7 to + 1.4 L, Fig. 3b), and PV reductions were ~ 5% smaller (95% CI -2 to -8%, Fig. 3c) compared with 60FR. In most endurance exercise scenarios, hypotonic sweat losses increase S_[Na_^+^_]_ and S_osm_, which is seen as an important change required to activate a cascade of physiological responses and ultimately accelerate fatigue (James et al. 2019). Here, however, S_[Na_^+^_]_ was stable over time and across trials, yet TT performance improved in 80FR (− 175 s; + 22 W). Taken together, these data indicate that smaller reductions in TBW and PV provide a plausible explanation for the performance benefit observed independent of changes in S_[Na_^+^_]_. This builds upon the consensus that BML ≥ 2% is ergolytic in the heat (Cheuvront et al. 2003; Sawka et al. 2007; James et al. 2019), suggesting that reductions in TBW and hypovolaemia of sufficient magnitude may affect performance in the absence of such osmotic changes. Although the independent contributions of additional fluid and Na⁺ to the observed changes in hydration and performance cannot be determined, the higher Na⁺ dose in 80FR may have contributed to greater intravascular fluid retention (Stachenfeld 2008), helping to attenuate PV reductions. Future work should match fluid intake across trials and compare personalised Na^+^ provision with a placebo, to isolate the effect of Na^+^. Another important question concerns whether Na^+^ ingestion during prolonged endurance exercise influences ad-libitum fluid intake, and thereby indirectly affects performance through altered drinking behaviour. Some evidence suggests that Na^+^ may stimulate fluid consumption and promote improved fluid balance in field-based endurance settings (Cosgrove and Black 2013; Del Coso et al. 2016). However, data from controlled laboratory studies conducted in hot conditions remain limited.

Post-preload, T_re_ was 0.30 °C lower in 80FR (95% CI -0.47 to -0.13 °C, Fig. 5a), despite no differences in WBSL, WBSR, LSR or sweat electrolytes during the preload, indicating that altered sweat responses were not responsible for attenuating the rise in T_re_ prior to the TT. Instead, the greater hypovolaemia in 60FR may have reduced SkBF (Nadel et al. 1980; Fortney et al. 1981; Montain and Coyle 1992; Sawka et al. 2001), supported by the lower T_sk_ during the TT in 60FR. Reductions in skin perfusion can compromise convective and radiative heat loss (Roberts and Wenger 1979; Sawka et al. 1984) and internal core-to-skin heat transfer (Nadel et al. 1980), potentially explaining the increase in body heat storage in 60FR. Our data build upon previous findings that diuretic-induced iso-osmotic hypohydration can increase T_core_ (Nadel et al. 1980), demonstrating that increased magnitude of exercise-induced hypohydration and hypovolaemia can also raise T_core_, even in the absence of changes in S_[Na_^+^_]_. However, hypertonicity may exacerbate these increases in T_core_ (Périard et al. 2021).

Despite comparable S_[Na_^+^_]_, thirst sensation was higher (Fig. 3d) and urine volume lower in 60FR, likely due to the exacerbated hypovolaemia. Reductions in PV of 8–10% can increase thirst, even without simultaneous changes in S_osm_ (Hughes et al. 2018), consistent with the ~ 10–11% ∆PV in 60FR. Slightly higher (though non-significant) baseline thirst in 60FR may also have contributed, as subsequent changes in thirst were similar between trials. Despite this, TT ad-libitum fluid intake was unaffected, suggesting that thirst effects were insufficient to increase voluntary fluid ingestion. Hypovolaemia can also reduce renal perfusion (Smith et al. 1952), which can lower glomerular filtration rate (Leatherby et al. 2021), independent of osmotic regulation. Hence, the lower urine volume in 60FR likely reflect changes in renal perfusion, rather than osmotic or hormonal changes.

Findings suggest that in hot conditions, cyclists should aim to replace sufficient fluid to limit BML to < 2% and consume personalised Na^+^ to maintain S_[Na_^+^_]_, thereby mitigating PV/TBW losses, lowering T_core_ and subsequently enabling higher power output. For athletes who struggle to maintain BML within 2% during prolonged efforts, consuming some additional Na^+^ above that required to maintain S_[Na_^+^_]_ and S_osm_ may be warranted, to stimulate thirst and voluntary fluid intake. Practitioners should note that drinking enough to prevent increases in S_[Na_^+^_]_ and S_osm_ alone may not optimise performance. Given that thirst is primarily driven by changes in S_[Na_^+^_]_ and S_osm_ (Stachenfeld 2008), these results suggest that “drinking to thirst” may not always be sufficient to prevent performance decrements during prolonged cycling exercise in the heat, as has been suggested by some researchers (Noakes 2010). Instead, hydration strategies should target BML < 1—2%, even when greater losses could theoretically occur without hyperosmolality. Examples of circumstances where it is predicted that an athlete could drink enough to theoretically dilute S_[Na_^+^_]_, but not enough to maintain BML to < 2% are when total fluid losses are ≥  ~ 5 L and whole-body sweat [Na^+^] is ≥  ~ 50 mmol∙L^−1^ (McCubbin 2025).

### Limitations

Participants were not fully blinded to hydration status, although differences in fluid provision were small (~ 70 mL every 15 min) and only 2/11 participants identified these differences, with no participants identifying differences in capsule NaCl content. Sweat patches were kept on for 30 min, rather than being assessed for saturation, which could underestimate sweat [Na^+^] due to hidromeiosis if the patch became oversaturated with sweat (Baker 2017). However, mean sweat [Na^+^] values fell within the standard deviation of normative values for endurance athletes (Barnes et al. 2019), suggesting hidromeiosis is unlikely to have significantly impacted results. In addition to this, [K^+^] values for all sweat samples were < 10 mmol∙L^−1^, indicating that leaching or sample evaporation/contamination were also unlikely to have occurred during sweat sampling (Baker 2017).

It should also be acknowledged that interpretation of ∆PV values may be influenced by posture (Reynolds et al. 2025), as the baseline sample was obtained in the supine position and subsequent samples were collected during cycling. However, sampling posture was identical across trials, ensuring valid between-trial comparisons. The absence of direct S_osm_ measurements may also be considered a limitation, given that body water regulation and compartmental distribution are primarily governed by osmotic stimuli (Verbalis 2003). However, S_[Na_^+^_]_ and S_[Cl_^−^_],_ which have by far the biggest influence on S_osm_, together accounting for ~ 80% of extracellular osmolality (Rowe 2018), were quantified, meaning the principal osmotic determinants were captured. Finally, the lack of direct measures of SkBF or indices of central fatigue limits mechanistic interpretation. These variables should be prioritised in future studies examining the independent effects of hypovolaemia and serum tonicity/electrolyte content.

Findings are specific to trained male cyclists under controlled laboratory heat stress and hence may not fully generalise to females, other populations, or field conditions with variable solar radiation, airflow, and hydration opportunities. The normalisation of work completed for the three participants whose trials were terminated early also introduces a potential limitation when comparing pacing strategies between trials.

## Conclusion

Replacing 60% of fluid losses during 3 h of cycling in the heat resulted in ~ 2.5% BML, greater hypovolaemia and higher T_re_, and was associated with impaired subsequent TT performance compared with 80% replacement (~ 1.5% BML). These differences occurred despite personalised Na^+^ ingestion maintaining comparable S_[Na_^+^_]_ between trials. Collectively, these findings indicate that inadequate fluid replacement resulting in hypovolaemia may impair thermoregulation and cycling performance in the heat, even when S_[Na_^+^_]_ remains stable.

## Data Availability

Data supporting these findings is available from the corresponding author upon reasonable request.

## Abbreviations

60FR: Sixty percent fluid replacement trial
80FR: Eighty percent fluid replacement trial
BM: Body mass
BML: Body mass loss
HR: Heart rate
LSR: Local sweat rate
LT_1_: First lactate threshold
Na^+^: Sodium
NaCl: Sodium chloride
PPO: Peak power output
PV: Plasma volume
SC: Sweat characterisation visit
S_[Cl_^−^_]_: Serum chloride concentration
S_[K_^+^_]_: Serum potassium concentration
SkBF: Skin blood flow
S_[Na_^+^_]_: Serum sodium concentration
S_osm_: Serum osmolality
TBW: Total body water
T_re_: Rectal temperature
T_sk_: Skin temperature
TT: Time-trial
U_osm_: Urine osmolality
V̇O_2max_: Maximum oxygen consumption
WBSL: Whole-body sweat loss
WBSR: Whole-body sweat rate
